# Lung function parameters improve prediction of VO_2peak_ in an elderly population: The Generation 100 study

**DOI:** 10.1371/journal.pone.0174058

**Published:** 2017-03-20

**Authors:** Erlend Hassel, Dorthe Stensvold, Thomas Halvorsen, Ulrik Wisløff, Arnulf Langhammer, Sigurd Steinshamn

**Affiliations:** 1 K.G. Jebsen Center of Exercise in Medicine, Department of Circulation and Medical Imaging, Norwegian University of Science and Technology, Trondheim, Norway; 2 Clinic of Thoracic and Occupational Medicine, St. Olavs Hospital, Trondheim University Hospital, Trondheim, Norway; 3 Department of Health Research, SINTEF Technology and Society, Trondheim, Norway; 4 Department of Public Health and General Practice, Norwegian University of Science and Technology, Trondheim, Norway; Charité—Universitätsmedizin Berlin, GERMANY

## Abstract

Peak oxygen uptake (VO_2peak_) is an indicator of cardiovascular health and a useful tool for risk stratification. Direct measurement of VO_2peak_ is resource-demanding and may be contraindicated. There exist several non-exercise models to estimate VO_2peak_ that utilize easily obtainable health parameters, but none of them includes lung function measures or hemoglobin concentrations. We aimed to test whether addition of these parameters could improve prediction of VO_2peak_ compared to an established model that includes age, waist circumference, self-reported physical activity and resting heart rate. We included 1431 subjects aged 69-77 years that completed a laboratory test of VO_2peak_, spirometry, and a gas diffusion test. Prediction models for VO_2peak_ were developed with multiple linear regression, and goodness of fit was evaluated. Forced expiratory volume in one second (FEV_1_), diffusing capacity of the lung for carbon monoxide and blood hemoglobin concentration significantly improved the ability of the established model to predict VO_2peak_. The explained variance of the model increased from 31% to 48% for men and from 32% to 38% for women (p<0.001). FEV_1_, diffusing capacity of the lungs for carbon monoxide and hemoglobin concentration substantially improved the accuracy of VO_2peak_ prediction when added to an established model in an elderly population.

## Introduction

Cardiorespiratory fitness is an indicator of cardiovascular health and is a good predictor of all-cause mortality [1]. Measurement of peak oxygen uptake (VO_2peak_) during an incremental work test is considered the best measure of cardiorespiratory fitness [2, 3]. Measured VO_2peak_ is a useful tool for risk stratification and is of interest when planning and evaluating medical treatment, surgery or rehabilitation [4, 5]. However, incremental work tests are not routinely used in most healthcare settings as they are time-consuming, costly and require trained personnel and expensive equipment, and are also contraindicated in some patients.

To avoid the disadvantages with incremental work tests, several non-exercise models to estimate VO_2peak_ have been developed [6–12]. The models have included easily obtainable measures such as sex, age, self-reported physical activity, resting heart rate, smoking history, BMI, waist circumference and body composition. Models of non-exercise estimation of VO_2peak_ have been shown to predict cardiovascular and all-cause mortality [13, 14].

VO_2peak_ is an important predictor of morbidity and mortality [15], and an indicator of functional decline in elderly [16]. With increasing age the mortality and morbidity of most diseases increase. Tools for prediction of VO_2peak_ without the need of an exercise test could be particularly useful for both clinical and research purposes in this age group.

VO_2peak_ reflects the maximal rate the body can take up oxygen from the surrounding air and utilize it to produce energy-rich substrate for biological functions. This process comprises several steps: air inspiration, diffusion of O_2_ over the alveolocapillary membrane, binding to blood hemoglobin, transport through the cardiovascular system and diffusion of oxygen from the blood into the muscle cells, and finally utilization of oxygen by the mitochondrial enzymes. While it is not possible to measure the functional capacity of the cardiovascular system without exercise testing, several of the other steps of the oxygen uptake and transport are easily measurable at rest. Spirometry measures the capacity of the respiratory system to transport air into and out of the lungs. Diffusion capacity testing measures the conductance of gas from the alveoli across the alveolocapillary membrane until binding to the hemoglobin in the erythrocytes. Spirometry data and blood hemoglobin concentration are usually available or easily obtainable in a general practice setting, while CO-diffusion data are frequently available in hospital settings. To our knowledge, measurements of pulmonary function have not previously been evaluated in non-exercise estimation models of VO_2peak_. Based on the physiological connection between oxygen uptake and these parameters, we hypothesize that they can improve a non-exercise estimation model of VO_2peak_.

We have previously shown that forced expiratory volume in 1 second (FEV_1_) and diffusing capacity of the lungs divided by alveolar volume (D_LCO_/VA) are associated with VO_2peak_ [17] in elderly. It is therefore relevant to include lung function indices as predictors in non-exercise estimation models of VO_2peak_ in elderly.

In a mostly healthy elderly population aged 69–77 years, we included spirometry data, hemoglobin values and lung diffusing capacity data in a previously validated non-exercise prediction model of VO_2peak_ developed by Nes et al [7]. We hypothesized that addition of these lung function indices would improve the prediction of VO_2peak_ in this population.

## Methods

Study subjects were obtained from the Generation 100 study with clinicaltrials.gov identifier NCT01666340. This is a randomized controlled study on the effects of an exercise intervention in elderly, previously described in detail [17, 18]. All participants gave written informed consent. Briefly, all persons born from 1^st^ January 1936 to 31^st^ December 1942 who were registered inhabitants of Trondheim municipality by the 1^st^ January 2012 (n = 6966) were invited to participate in a randomized, controlled trial aiming to study the effect of an exercise intervention on morbidity and mortality. Subjects with conditions or test results indicating that high intensity exercise could be unsafe were excluded. The present study uses baseline, pre-randomization data from the Generation 100 study. Baseline testing were performed as previously described and included symptom-limited test of VO_2peak_; spirometry (pre-bronchodilator values), lung diffusion capacity (Sensormedics Vmax22 Encore, CareFusion, San Diego, USA) in accordance with the ATS/ERS standardized procedures[19, 20]. VO_2peak_ was measured by an incremental work test on a treadmill using the gas analyzer Oxycon Pro (Erich Jaeger, Hoechberg, Germany, n = 67) or Cortex MetaMax II (Leipzig, Germany, n = 1364). After a warm-up period, the work load was increased by 1 km/h or 2% inclination every 1 to 2 minutes until exhaustion. Tests were aborted if subjects reported to have chest pain, nausea or dizziness. Subjects previously having aborted testing due to such symptoms and also those with previously diagnosed heart disease were supervised by a trained physician during testing with monitoring of blood pressure and ECG as recommended [21]. Every 10 seconds the gas analyzers reported average values from the last 30 seconds, and VO_2peak_ was calculated as the average of the three highest consecutive VO_2_-values. Ventilation, respiratory exchange ratio, heart rate and rated perceived exertion at peak work were registered, but not used as criterions for defining VO_2peak_. Physical activity index (PAI) was calculated from self-reported physical activity as previously described [7]. Peripheral capillary oxygen saturation (SpO_2_) at rest was measured (Nonin 8500 Pulse oximeter, Nonin Medical Inc., Plymouth, MN, USA) and blood samples were analyzed for hemoglobin concentration. The Generation 100 Study and the present sub-study were approved by the Regional Committee for Medical Research Ethics (REK 2012/381 B) and all participants gave written informed consents.

### Statistical analysis

Predicted values for gas diffusion and spirometry data were calculated from relevant reference equations [22, 23]. Breathing reserve (BR) was calculated as BR = 1-(minute volume at peak exercise/(FEV_1_x40))[24]. Multiple linear regression analyses with VO_2peak_ (mL·kg^-1^·min^-1^) as the dependent variable were performed separately for men and women. In the first model, the previously used predictors age (rounded to nearest year), PAI, resting heart rate (RHR) and waist circumference (WC) [7] were included, whilst in two further models hemoglobin and FEV_1_, and D_LCO_/VA were added. D_LCO_/VA was not corrected for hemoglobin. Forced vital capacity (FVC), FEV_1_/FVC, SpO_2_ and D_LCO_ and VA as separate variables were also tested as predictors, but were not included in the final model. Model assumptions were tested using residuals vs fitted values plots. Due to concerns about heteroscedasticity robust estimation of standard errors was used. Collinearity between the variables was assessed by tolerance and variance inflation factor. Due to concerns about non-normal distributions and to assess the internal validity of the models bootstrapping was performed with 10000 randomly drawn samples with replacement and *n* equal to the total sex-specific cases. This was used to calculate bootstrapped 95% bias-corrected and accelerated confidence intervals for the regression coefficients. The ability of the model to predict a VO_2peak_ in the lower tertile of measured values was evaluated by ROC curves with SigmaPlot 12.0 (Systat, San Jose, CA, USA). All other analyses were performed with IBM SPSS Statistics 23 (New York, USA) or Stata 13.1 (StataCorp, Texas, USA).

## Results

**Fig 1:**
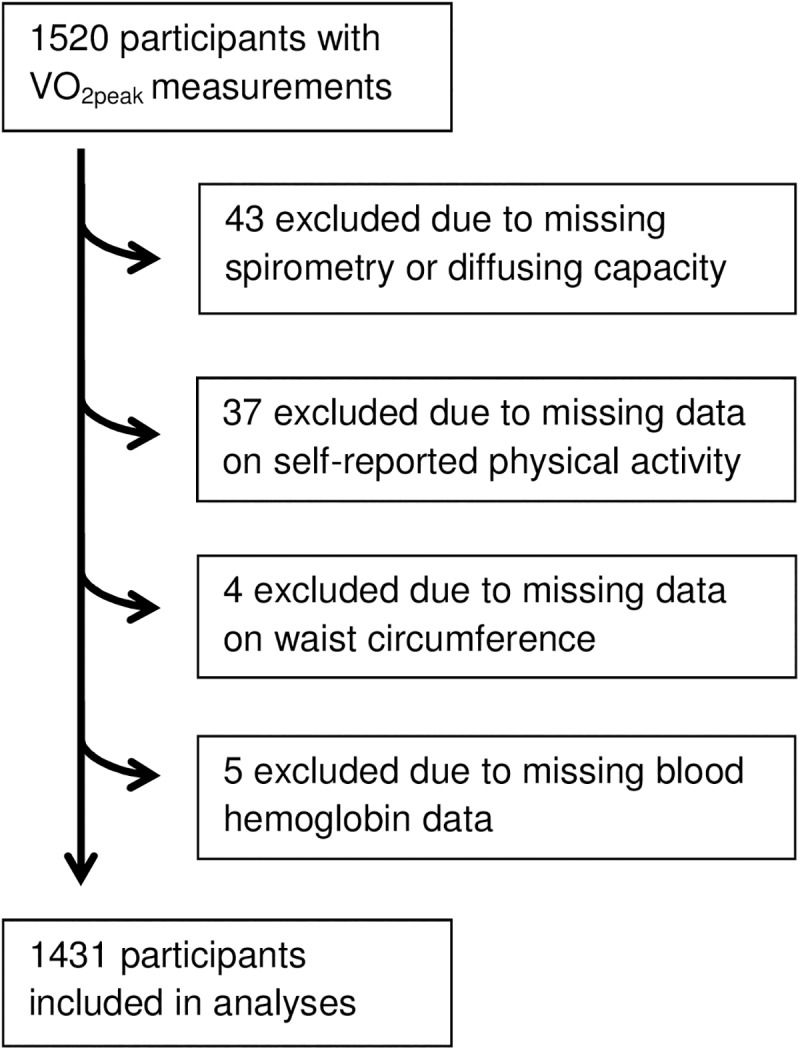
Flowchart showing participant excluded due to various missing data.

Valid VO_2peak_ measurements were obtained from 1520 participants. Of these 43, 37, 4 and 5 individuals were excluded due to missing data on spirometry or D_LCO_-tests, self-reported physical activity, waist circumference and hemoglobin, respectively, giving 1431 cases eligible for analysis, see flowchart (Fig 1). About 8% of both men and women were current smokers whereas 50% of men and 37% of women were former smokers (Table 1). Mean FEV_1_ and FVC were respectively 94% and 103% of predicted values for men and 103% and 111% for women. In the never-smoking sub-sample the corresponding percentages were 97% and 104% for men and 105% and 112% for women. Mean peripheral saturation was 97% percent for both men and women. Plots showing distribution of VO_2peak_ and added predictors are shown in S1 File.

**Table 1:** Descriptive statistics for participants.

	Men (n = 722)	Women (n = 709)
Age (years)	72.8±2.0	72.9±2.1
Height (cm)	176.9±5.8	163.4±5.2
Weight (kg)	82.7±11.5	68.1±10.8
Body mass index (kg/m^2^)	26.4±3.3	25.5±3.7
Waist circumference (cm)	98.2±9.6	89.9±10.8
Smoking status (n)		
Current smoker	58 (8.1%)	60 (8.6%)
Former smoker	361 (50.3%)	257 (36.8%)
Never smoker	299 (41.6%)	382 (54.6%)
Resting heart rate (bpm)	62.8±11.2	66.9±9.9
FEV_1_		
(liters)	3.13±0.61	2.24±0.36
(% of predicted)	93.7±16.8	102.6±15.8
<80% of predicted (n)	123 (17.0%)	52 (7.3%)
FVC		
(liters)	4.35±0.73	3.06±0.46
(% of predicted)	103.2±15.1	111.0±15.3
FEV_1_/FVC		
(%)	72.0±8.1	73.5±6.6
ratio<0.7 (n)	228 (31.6%)	176 (24.8%)
D_LCO_		
(mmol·min^-1^·kPa^-1^)	8.99±1.74	6.71±1.14
(% of predicted)	93.1±16.7	86.1±13.5
VA		
(liters)	6.60±0.94	4.82±0.64
(% of predicted)	101.1±12.2	98.9±12.0
D_LCO_/VA		
(mmol·min^-1^·kPa^-1^·L^-1^)	1.37±0.23	1.40±0.20
(% of predicted)	93.6±18.7	84.8±12.0
<75% of predicted (n)	110 (15.2%)	131 (18.5%)
Resting SpO_2_ (%)	96.8±1.4	97.1±1.4
Hemoglobin (g/dL)	15.0±1.1	13.9±0.9
Measurements obtained during exercise tests:		
VO_2peak_(mL·kg^-1^·min^-1^)	31.3±6.8	26.3±4.9
Peak heart rate (bpm)	157±18	157±15
Max treadmill speed (km/h)	6.0±1.3	5.2±0.9
Max treadmill inclination (%)	12.4±4.1	11.7±3.3
RER (ratio)	1.14±0.09	1.10±0.09
Breathing reserve (%)	21.9±16.7	30.8±13.2
Breathing reserve<15% (n)	211 (29.5%)	79 (11.3%)

Abbreviations: VO_2peak_ – peak oxygen uptake, FEV_1_ – forced expiratory volume in 1 second, FVC – forced vital capacity, D_LCO_ – diffusing capacity of the lung for carbon monoxide, VA – alveolar volume, SpO_2_ – peripheral capillary oxygen saturation, RER – respiratory exchange ratio at peak exercise. Values given as mean±standard deviation or n (column percentage).

Compared to the basic model with age, PAI, WC and RHR, additional inclusion of FEV_1_ and hemoglobin increased the explained variance (adjusted R^2^) of measured VO_2peak_ from 31% to 41% for men and from 32% to 34% for women (Table 2). Further addition of D_LCO_/VA increased the explained variance to 48% and 38% for men and women, respectively. Basic models expanded with only one predictor (FEV_1_, D_LCO_/VA or hemoglobin) are shown in S2 File. The prediction equations (Table 3) had tolerance > 0.8 and variance inflation factor < 1.2 indicating no multicollinearity issues. FVC, FEV_1_/FVC and D_LCO_ and VA as separate variables were also tested in the model, but their additional contributions to explained variance of VO_2peak_ were negligible and did also introduce multicollinearity problems. SpO_2_ did not improve prediction. Comparison of the results from the bootstrap analyses with that of the normal regression analyses revealed only marginal differences. Bootstrapped confidence intervals and standard deviations and the same statistics from the normal regression analyses yielded the same conclusions attesting to the generalizability of the model to similar populations.

**Table 2:** Summary of multiple linear regressions models predicting VO_2peak._

		Men				Women			
Model	Predictors	R	Adj.R^2^	R^2^ change	SEE	R	Adj.R^2^	R^2^ change	SEE
1	Age, PAI, WC, RHR	0.56	0.31		5.61	0.57	0.32		4.04
2	Age, PAI, WC, RHR, FEV_1_, Hb	0.64	0.41	0.10***	5.21	0.59	0.34	0.03***	3.97
3	Age, PAI, WC, RHR, FEV_1_, Hb, D_LCO_/VA	0.70	0.48	0.07***	4.87	0.62	0.38	0.03***	3.87

Abbreviations: R – multiple correlation coefficient, SEE – standard error of estimate, PAI – Physical activity index calculated from Nes et al. [7], WC – waist circumference, PAI – Physical activity index, RHR – resting heart rate, FEV_1_ – forced expiratory volume in 1 second, Hb – blood hemoglobin concentration, D_LCO_/VA – diffusing capacity divided by alveolar volume.

*** p<0.001 (F-test) compared to previous step.

**Table 3:** Description of prediction models.

	Men			Women		
Variable	β	Bootstrapped 95%CI for β		β	Bootstrapped 95%CI for β	
Model 1 predictors: Age, PAI, WC and RHR						
Intercept	97.825***	(80.6, 115.0)		70.108***	(58.9, 80.9)	
Age	-0.458***	(-0.646, -0.269)		-0.306***	(-0.436, -0.164)	
PAI	0.172***	(0.125, 0.220)		0.123***	(0.092, 0.157)	
WC	-0.322***	(-0.368, -0.280)		-0.200***	(-0.228, -0.172)	
RHR	-0.050*	(-0.090, -0.007)		-0.068***	(-0.097, -0.039)	
Model 2 predictors: Age, PAI, WC, RHR, Hb and FEV_1_						
Intercept	64.893***	(46.5, 83.0)		56.205***	(44.2, 68.7)	
Age	-0.331***	(-0.518, -0.143)		-0.251***	(-0.391, -0.107)	
PAI	0.150***	(0.107, 0.194)		0.120***	(0.090, 0.154)	
WC	-0.320***	(-0.361, -0.284)		-0.199***	(-0.229, -0.169)	
RHR	-0.056**	(-0.094, -0.016)		-0.069***	(-0.097, -0.041)	
FEV_1_	2.967***	(2.289, 3.681)		2.041***	(1.199, 2.814)	
Hb	0.989***	(0.615, 1.382)		0.385*	(0.044, 0.728)	
Model 3 predictors: Age, PAI, WC, RHR, Hb, FEV_1_ and D_LCO_/VA						
Intercept	47.886***	(30.8, 65.3)		45.847***	(34.0, 58.4)	
Age	-0.177*	(-0.353, -0.009)		-0.178*	(-0.321, -0.038)	
PAI	0.131***	(0.092, 0.171)		0.114***	(0.084, 0.148)	
WC	-0.342***	(-0.379, -0.310)		-0.211***	(-0.240, -0.183)	
RHR	-0.055***	(-0.090, -0.021)		-0.068***	(-0.095, -0.040)	
FEV_1_	3.266***	(2.639, 3.888)		2.447***	(1.624, 3.233)	
Hb	0.693***	(0.305, 1.066)		0.287	(-0.057, 0.638)	
D_LCO_/VA	8.485***	(6.723, 10.190)		4.635***	(3.101, 6.156)	

Abbreviations: β – regression coefficient, β-weights – standardized coefficient, PAI – Physical activity index calculated from Nes et al. [7], WC – waist circumference, PAI – Physical activity index, RHR – resting heart rate, FEV_1_ – forced expiratory volume in 1 second, Hb – blood hemoglobin concentration, D_LCO_/VA – diffusing capacity divided by alveolar volume.

*p<0.05

**p<0.01

***p<0.001 (non-bootstrapped).

Description of prediction models with regression coefficients and bootstrapped confidence intervals. VO_2peak_ is dependent variable.

**Fig 2:**
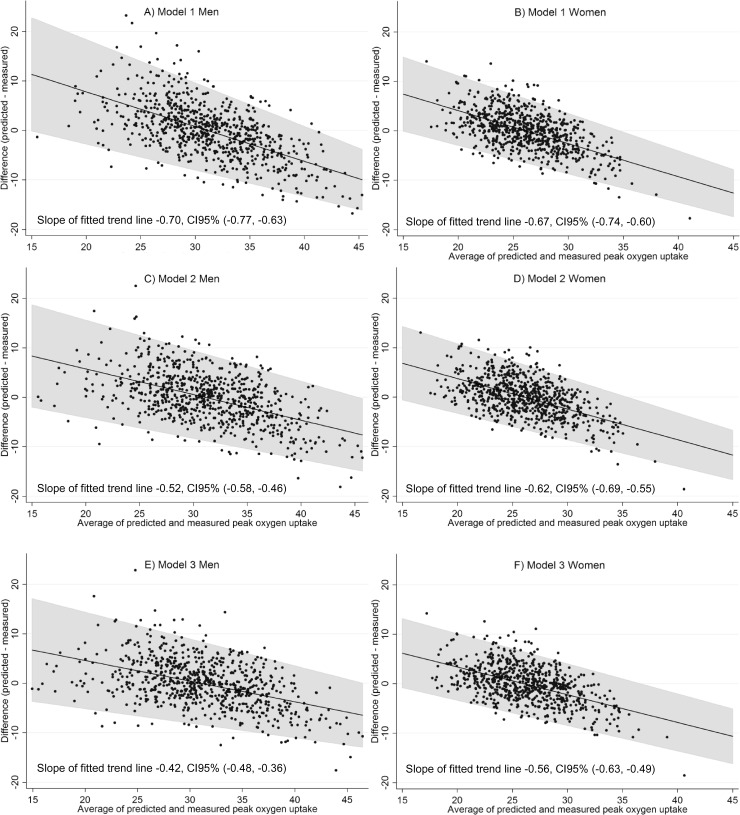
Bland-Altman plots for predictions models.

Bland-Altman plots (Fig 2) show that VO_2peak_ is overestimated for low levels of VO_2peak_, and underestimated for high levels. This over- and underestimation is attenuated when lung function and hemoglobin are added to the models. This attenuation is evident by the reduction in the slope of the trend lines in the Bland-Altman plots as these parameters are added in the models.

Difference between predicted and measured VO_2peak_ is plotted against the average of the predicted and measured VO_2peak_. A line of best fit is plotted to show trends. Slope and 95% confidence interval for slope for this line is given. Shaded areas represent 95% limits of agreement.

**Fig 3:**
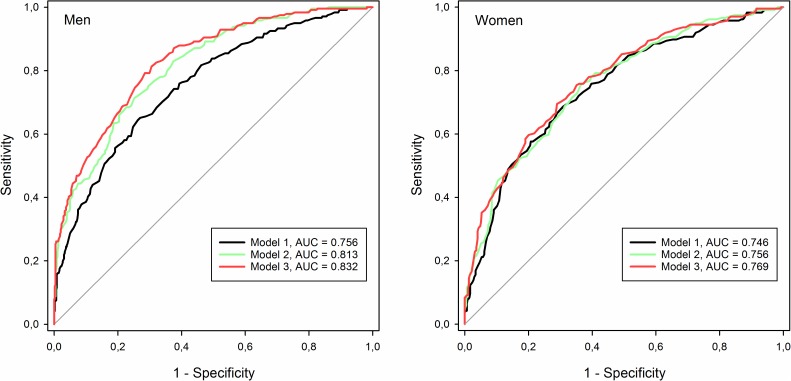
Performance of models identifying subjects with VO_2peak_ in lower tertile.

To better visualize how adding parameters to the models change their predictive performance, the ability of the models to correctly identify subjects in the lower tertile of measured VO_2peak_ was illustrated using Receiver Operating Characteristic (ROC) curves. Adding hemoglobin and lung function improved the ability of the models to identify men in the lower tertile of VO_2peak_, as is shown by increased area under the ROC curve, but little effect was seen for women (Fig 3).

ROC curves for detecting subjects in the lower tertile of measured VO_2peak_ (<28.1 ml·kg^-1^·min^-1^ for men, <23.9 ml·kg^-1^·min^-1^ for women). Significance of change in area under curve (AUC) for men: model 1 vs. model 2 p<0.001, model 1 vs. model 3 p<0.001, model 2 vs. model 3 p = 0.052; women: model 1 vs. model 2 p = 0.108, model 1 vs. model 3 p = 0.009, model 2 vs. model 3 p = 0.052 (chi-square test).

## Discussion

In this study we found that hemoglobin and lung function measurements can be used to improve a previously developed non-exercise model of VO_2peak_ prediction in elderly individuals. A new prediction model containing FEV_1_, hemoglobin and D_LCO_/VA have been developed in this large study sample aged 69–77 years.

The explained variances (adjusted R^2^) estimated by the present prediction models (0.48 for men and 0.38 for women) are low compared with other comparable studies that have reported values in the range of 0.56–0.74 [7–11]. This can largely be explained by the relatively homogenous study group in general and especially the narrow age span with corresponding less contribution of age to the explained variance. The accuracy of the predictions evaluated with standard error of the estimate for VO_2peak_ (SEE) in our model show equivalent or better predictions (4.87 for men and 3.87 for women) compared to other models that have reported error values in the range of 4.7–5.7 [7–11].

The clinical usefulness of VO_2peak_ prediction models is much dependent on their ability to identify individuals with low fitness. Those with low fitness have not only increased risk for disease and death, but also the greatest potential for improvements in their fitness level from exercise interventions. Our results suggest that the ability of non-exercise estimation models of VO_2peak_ to correctly identify elderly subject with low fitness can be improved by adding measurements of lung function and hemoglobin to the prediction models.

Our models show that FEV_1_ and D_LCO_/VA are, at least for men, potent determinants for VO_2peak_. Pulmonary function has to our knowledge not previously been evaluated in non-exercise prediction models of VO_2peak_, maybe due to a conception that the lungs have a spare capacity and are not limiting exercise capacity in healthy subjects.

There are few studies on lung diffusing capacity in the elderly, and to our knowledge, the present data constitute the largest data set gathered. Compared with predicted values from equations developed in a healthy never-smoking Spanish population aged 65–85 [22], measured mean D_LCO_ and D_LCO_/VA were about 7% lower than predicted for men and 15% lower than predicted for women. For the never-smoking sub-sample corresponding figures were 2% and 13% lower in men and women, respectively. Measured mean for VA were very close to the predicted mean.

The increase in the explained variance from adding lung function measurements and hemoglobin to the prediction models is greater for men than for women. The improvements in prediction for men are also evident from the ROC curves in Fig 3, but for women the effects of adding these variables are marginal. One of the possible explanations for this sex-difference is that a larger proportion of the men seem to be ventilatory limited during exercise. Reaching minute ventilation constituting more than 85% of the maximal voluntary ventilation during exercise is regarded as a sign of ventilatory limitation. Among our study subjects almost three times as many men as women reached this threshold indicating that they might have their maximal exercise capacity limited by a relatively low ventilatory function. The distribution of measured lung function is also different between the sexes. Men have considerably higher standard deviations for all measured pulmonary function parameters compared to women. The higher spread in lung function for men gives lung function a higher potential for explaining variance of VO_2peak_ in men than in women.

The strengths of this study are the population-based design and the extensive testing of pulmonary function and directly measured VO_2peak_ in a large sample of elderly. No other non-exercise VO_2peak_ prediction model have been developed or validated in a larger population of elderly. Bootstrapped confidence intervals and standard deviations and the same statistics from the normal regression analyses yielded the same conclusions, attesting to the generalizability of the model to similar populations. Few of the study subjects had severe cardiopulmonary restrictions, so the models cannot be assumed to apply to such individuals. The study subjects were invited to participate in an exercise intervention study and it is possible that this have led to selection bias favoring fit individuals or those with a special interest for exercise. Ventilatory function and gas diffusing capacity is reduced with age, and while reduced lung function may limit maximal oxygen uptake in elderly, this may not be the case for younger subjects. The prediction models were developed in a relatively fit and healthy population aged 69–77 years. Before the models are applied beyond this age span or for less active individuals, or for individuals with various health conditions, they should be validated against such groups.

## Conclusions

We have shown that a validated prediction model of VO_2peak_ can be significantly improved by adding hemoglobin, pre-bronchodilator FEV_1_ and D_LCO_/VA measurements in an elderly population. Especially in men, the lung function parameters are shown to be important predictors of cardiovascular fitness. The developed prediction equations may be useful in some clinical or research settings where incremental exercise tests are considered impractical or too resource demanding. Although these models give a rough estimate of cardiorespiratory fitness, it must be emphasized that direct measurement of oxygen uptake during an exercise test is still a far superior method.

## Supporting information

S1 File Supplemental plots. (PDF)

S2 File Supplemental tables. (PDF)
