# Supplementary material for: Lung function parameters improve prediction of VO2peak in an elderly population: The Generation 100 study
Source: PLoS One. 2017 Mar 20;12(3):e0174058. doi: 10.1371/journal.pone.0174058 (PMC5358855; doi:10.1371/journal.pone.0174058)
Supplement: S1 File — (PDF) [file pone.0174058.s001.pdf]

S1 File. Boxplots and histograms with normal curves for dependent and added independent variables

Dashed line in box-plots represent mean and bold line represent median.

VO_{2peak}

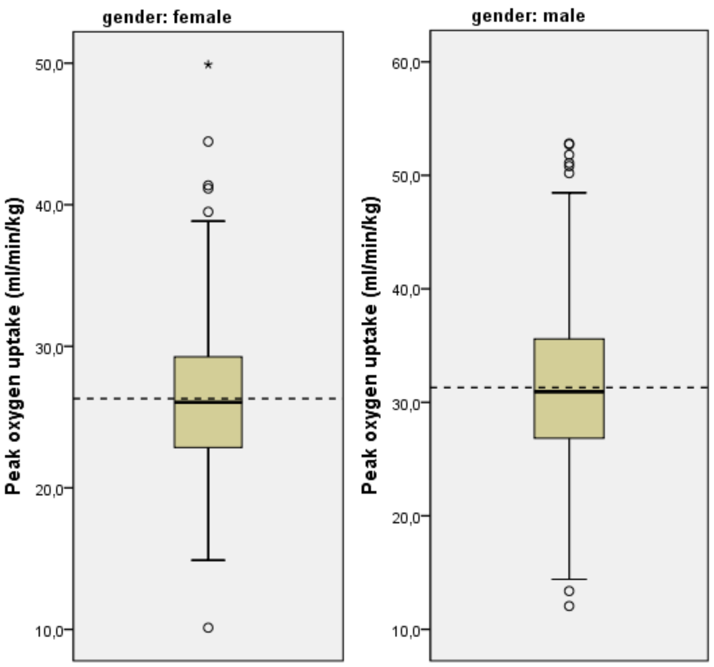

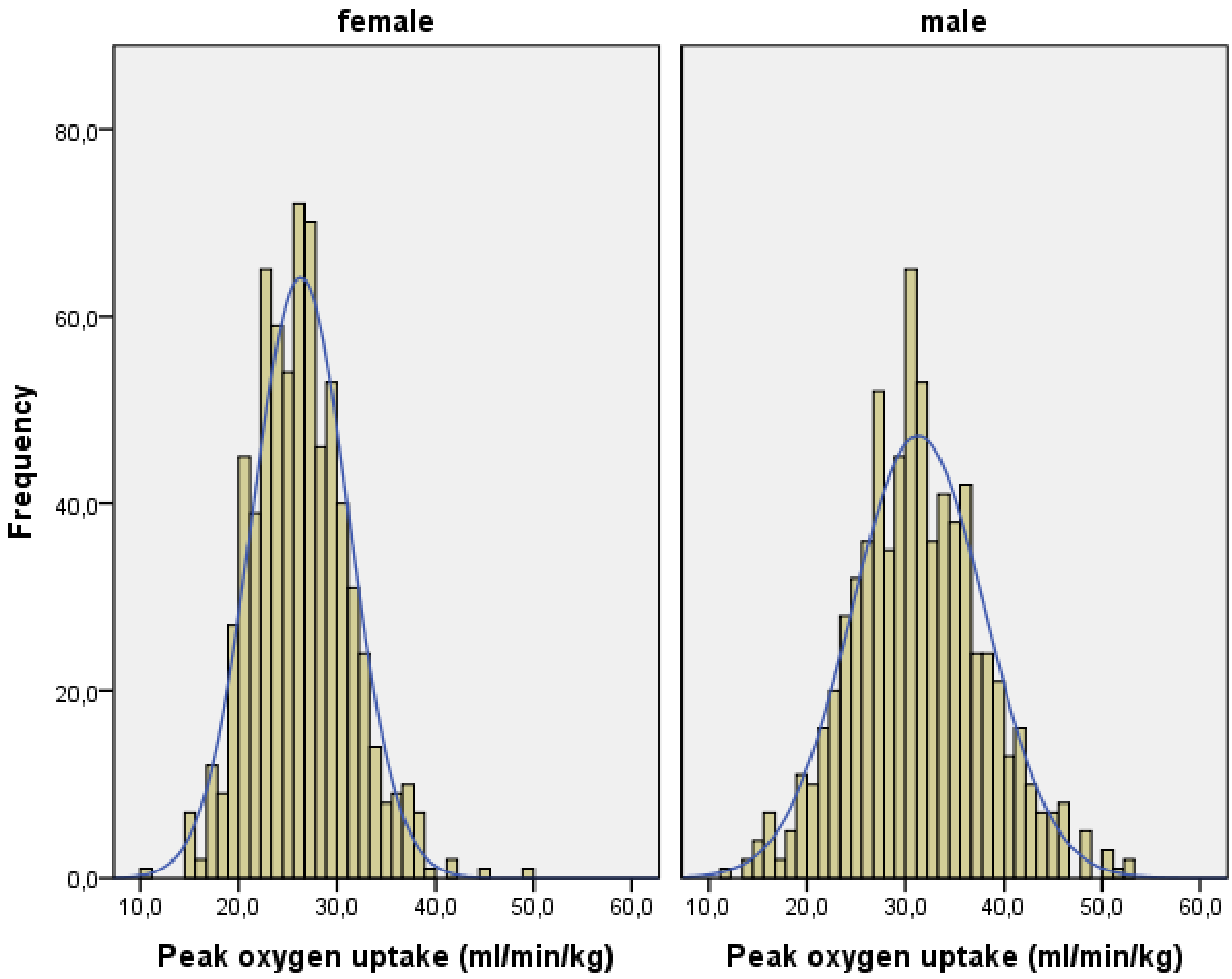

FEV_1

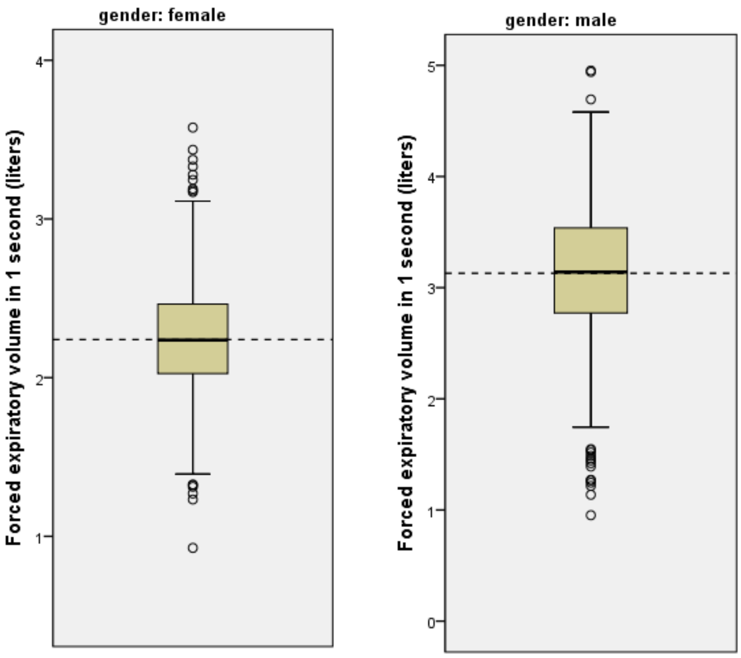
gender: female
gender: male
Forced expiratory volume in 1 second (liters)
Forced expiratory volume in 1 second (liters)

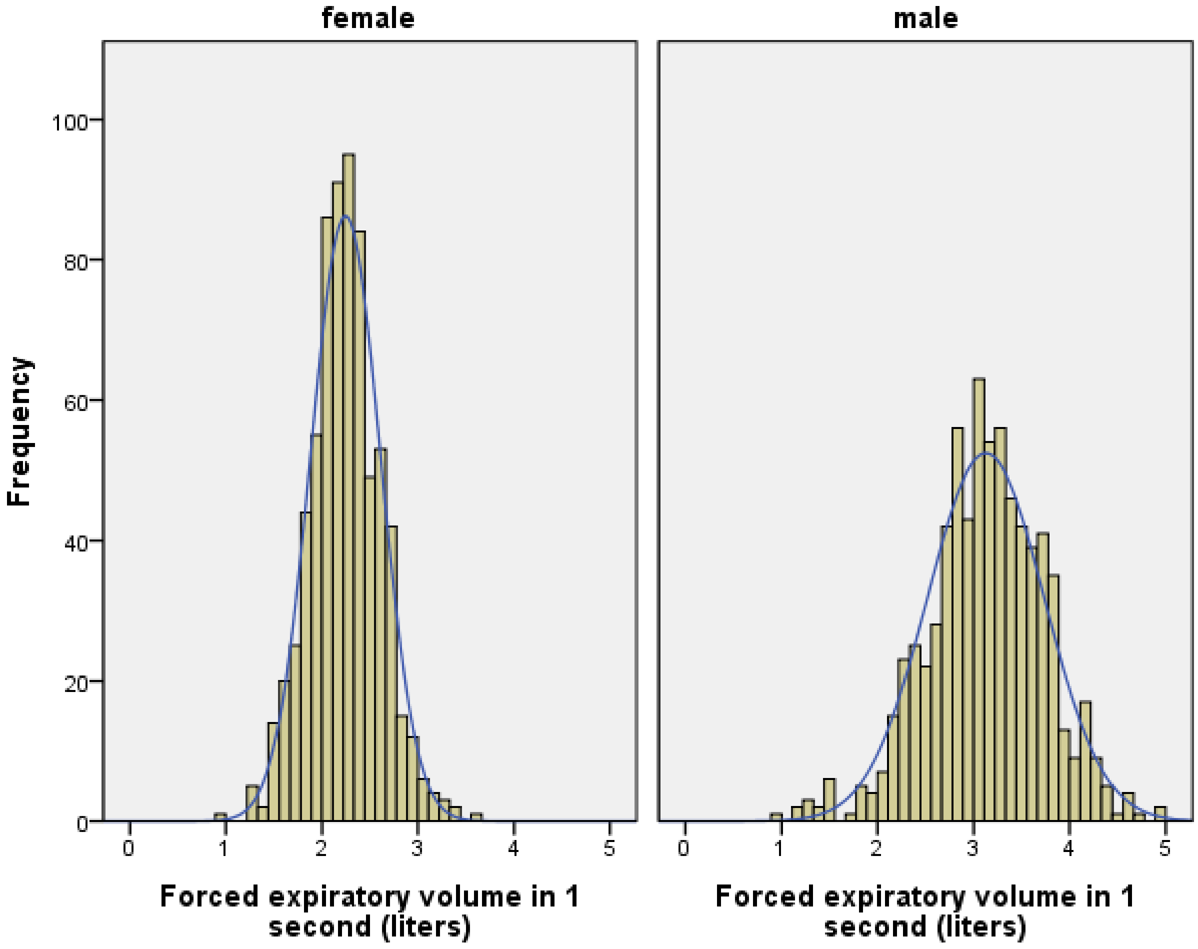
female
male
Frequency
Forced expiratory volume in 1 second (liters)
Forced expiratory volume in 1 second (liters)

Hemoglobin

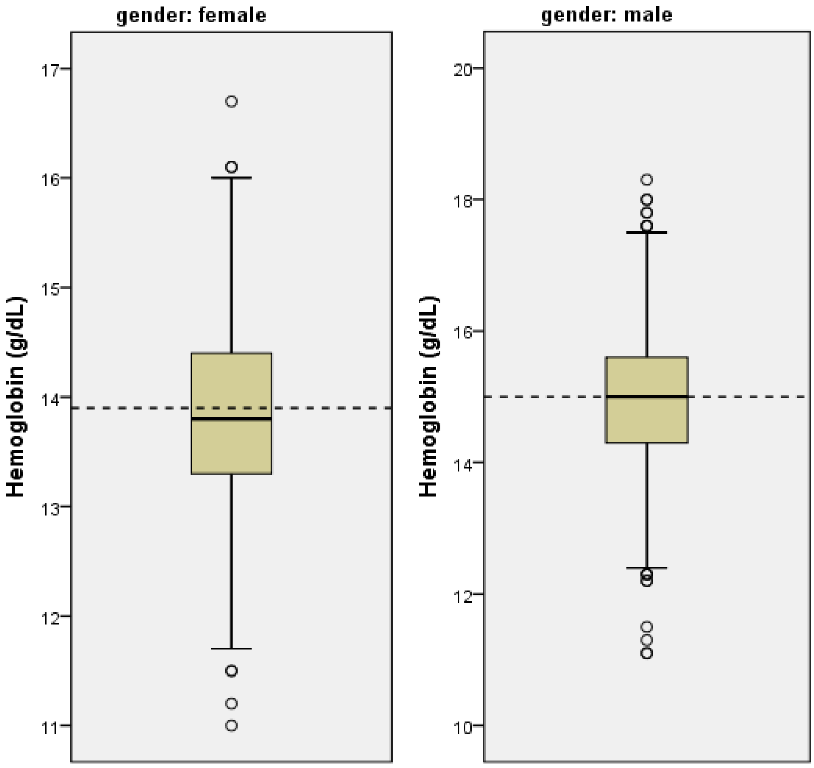
gender: female
gender: male
Hemoglobin (g/dL)

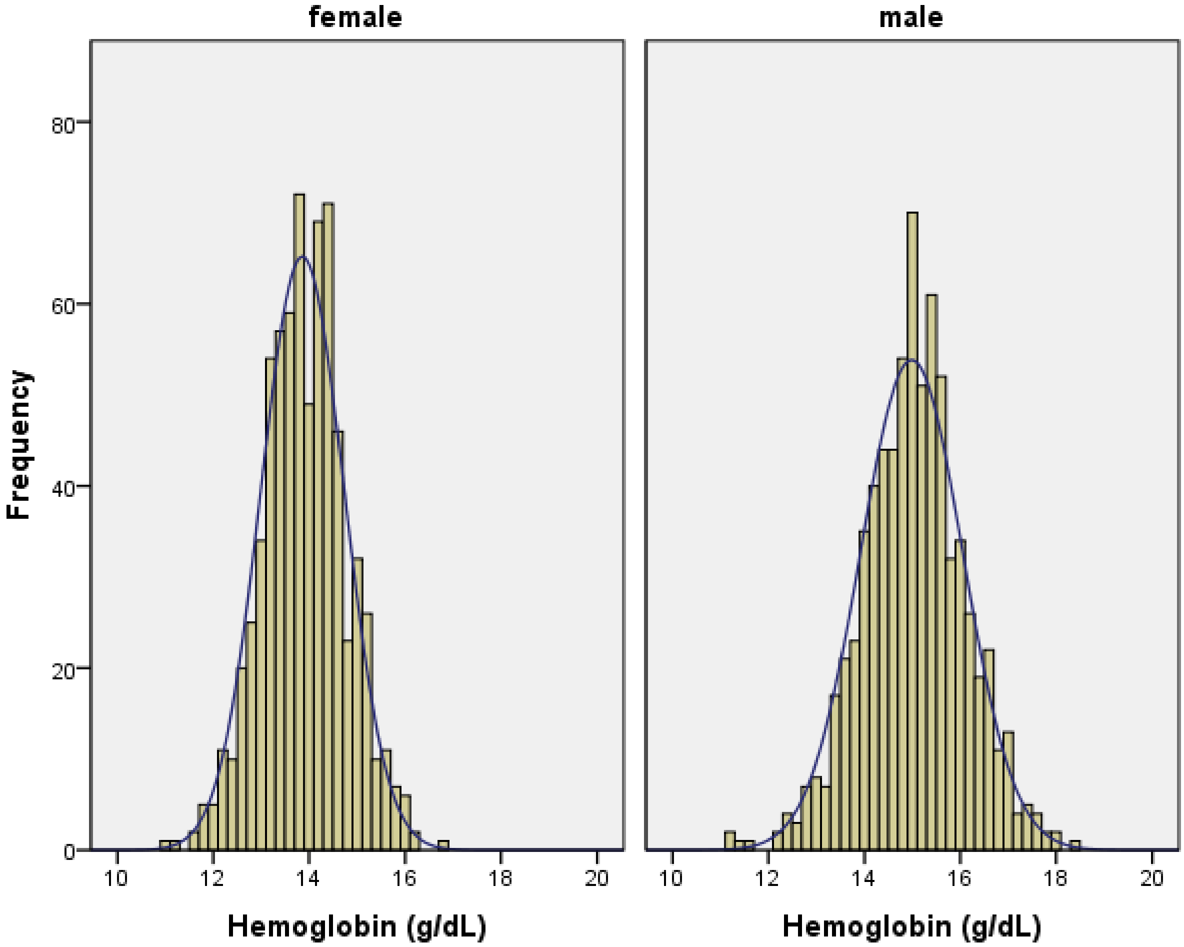
female
male
Frequency
Hemoglobin (g/dL)

D_{LCO}/VA

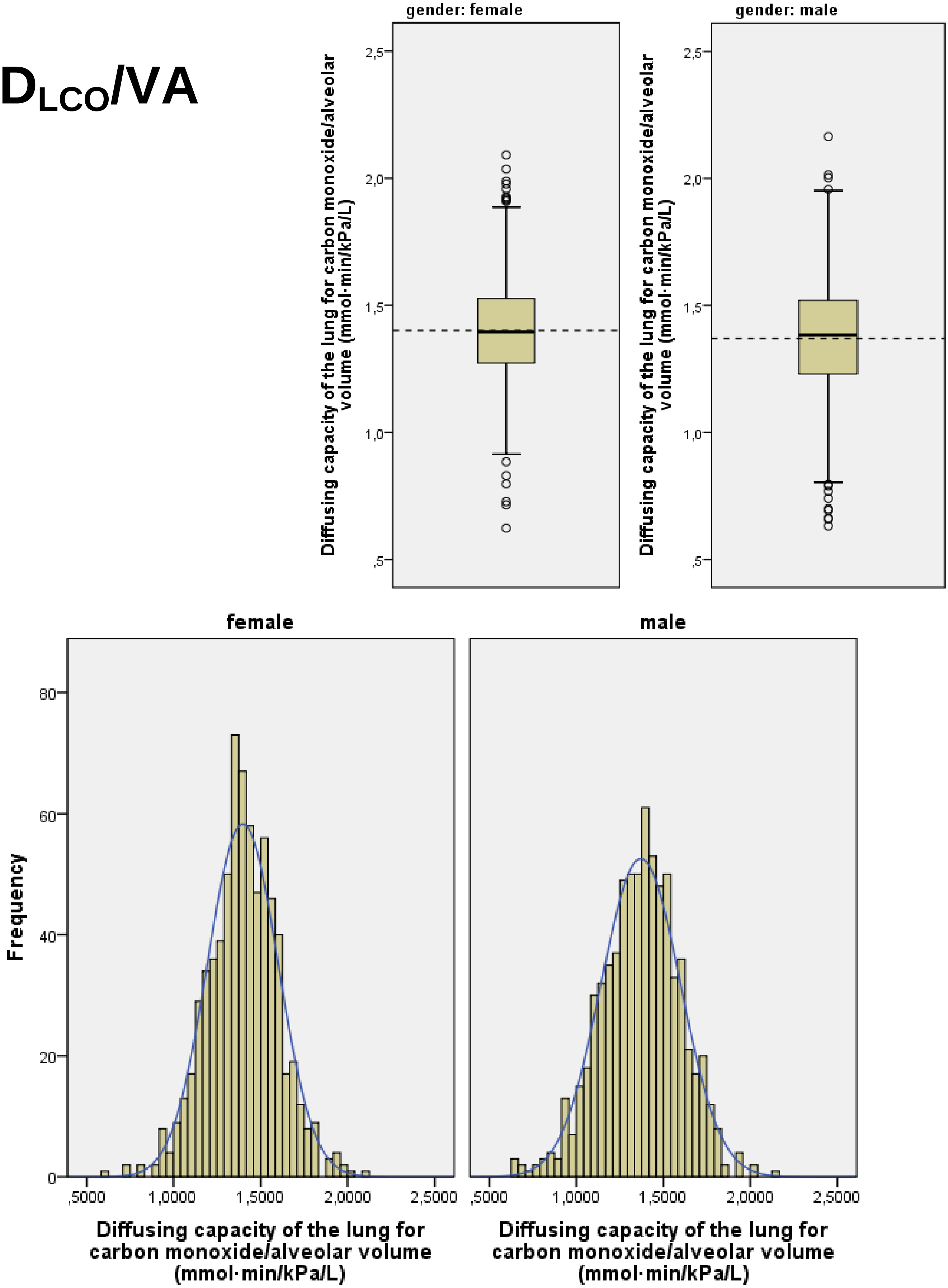

gender: female
gender: male
Diffusing capacity of the lung for carbon monoxide/alveolar volume (mmol·min/kPa/L)
2,5
2,0
1,5
1,0
,5
female
male
Frequency
80
60
40
20
0
,5000
1,0000
1,5000
2,0000
2,5000
Diffusing capacity of the lung for carbon monoxide/alveolar volume (mmol·min/kPa/L)
