# Supplementary material for: Lung function parameters improve prediction of VO2peak in an elderly population: The Generation 100 study
Source: PLoS One. 2017 Mar 20;12(3):e0174058. doi: 10.1371/journal.pone.0174058 (PMC5358855; doi:10.1371/journal.pone.0174058)
Supplement: S2 File — (PDF) [file pone.0174058.s002.pdf]

S2 File. Description of regression models with FEV1, Hb and DLCO/VA added separately

Table A showing summary statistics of multiple linear regressions models predicting $VO_{2peak.}$Table B showing regression coefficients of models. Abbreviations: WC – waist circumference, RHR – resting heart rate, Act – Self-reported leisure time activity,FEV1 – Forced expiratory volume in 1 second, Hb – blood hemoglobin concentration, DLCO/VA – diffusing capacity of the lungs for carbon monoxide/estimated alveolar volume.

TABLE A

Independent variables	R	Adjusted R^2	Std. Error of the Estimate
Men:			
Age, Act, WC, RHR	.564	.318	5.6143
Age, Act, WC, RHR, FEV1	.626	.392	5.3059
Age, Act, WC, RHR, Hb	.589	.346	5.5003
Age, Act, WC, RHR, DLCO/VA	.625	.391	5.3102
Age, WC, RHR, Act, Hb, FEV1, DLCO/VA	.699	.484	4.8704
Women:			
Age, WC, RHR, Act	.569	.320	4.0415
Age, WC, RHR, Act, FEV1	.588	.341	3.9782
Age, WC, RHR, Act, Hb	.573	.323	4.0324
Age, WC, RHR, Act, DLCO/VA	.592	.345	3.9653
Age, WC, RHR, Act, Hb, FEV1, DLCO/VA	.619	.378	3.8669

TABLE B

Model components	B-coefficients men	B-coefficients women
Intercept	77.698	61.281
Resting_HR	-.048	-.066
WC (cm)	-.297	-.194
activityscore	.155	.122
Age	-.348	-.257
FEV1	3.077	2.023
Intercept	82.819	65.340
Resting_HR	-.059	-.072
WC (cm)	-.347	-.206
activityscore	.166	.121
Age	-.436	-.300
hemoglobin	1.097	.367
Intercept	79.244	61.547
Resting_HR	-.052	-.068
WC(cm)	-.353	-.213
activityscore	.153	.117
age	-.313	-.249
DLCO/VA	8.253	4.029
